# Identifying major depressive disorder with associated sleep disturbances through fMRI regional homogeneity at rest

**DOI:** 10.1186/s12888-023-05305-7

**Published:** 2023-11-07

**Authors:** Dan Lv, Yangpan Ou, Dan Xiao, Huabing Li, Feng Liu, Ping Li, Jingping Zhao, Wenbin Guo

**Affiliations:** 1 Department of Psychiatry, National Clinical Research Center for Mental Disorders, and National Center for Mental Disorders, The Second Xiangya Hospital of Central South University, Changsha, 410011 Hunan China; 2 Department of Psychiatry, Qiqihar Medical University, Qiqihar, 161006 Heilongjiang China; 3 Department of Health and Medicine, Harbin Institute of Technology, Harbin, 151001 Heilongjiang China; 4 Department of Radiology, The Second Xiangya Hospital of Central South University, Changsha, 410011 Hunan China; 5 Department of Radiology, Tianjin Medical University General Hospital, Tianjin, 300000 China

**Keywords:** Major depressive disorder, Sleep disturbances, Regional homogeneity, Neuroimaging diagnostic biomarker

## Abstract

**Background:**

Anomalies in regional homogeneity (ReHo) have been documented in patients with major depressive disorder (MDD) and sleep disturbances (SDs). This investigation aimed to scrutinize changes in ReHo in MDD patients with comorbid SD, and to devise potential diagnostic biomarkers for detecting sleep-related conditions in patients with MDD.

**Methods:**

Patients with MDD and healthy controls underwent resting-state functional magnetic resonance imaging scans. SD severity was quantified using the 17-item Hamilton Rating Scale for Depression. Subsequent to the acquisition of imaging data, ReHo analysis was performed, and a support vector machine (SVM) method was employed to assess the utility of ReHo in discriminating MDD patients with SD.

**Results:**

Compared with MDD patients without SD, MDD patients with SD exhibited increased ReHo values in the right posterior cingulate cortex (PCC)/precuneus, right median cingulate cortex, left postcentral gyrus (postCG), and right inferior temporal gyrus (ITG). Furthermore, the ReHo values in the right PCC/precuneus and ITG displayed a positive correlation with clinical symptoms across all patients. SVM classification results showed that a combination of abnormal ReHo in the left postCG and right ITG achieved an overall accuracy of 84.21%, a sensitivity of 81.82%, and a specificity of 87.50% in identifying MDD patients with SD from those without SD.

**Conclusion:**

We identified disrupted ReHo patterns in MDD patients with SD, and presented a prospective neuroimaging-based diagnostic biomarker for these patients.

**Supplementary Information:**

The online version contains supplementary material available at 10.1186/s12888-023-05305-7.

## Introduction

Major depressive disorder (MDD) is one of the most prevalent mental disorders, with a global prevalence of 4.4% [1, 2]. More than 300 million people suffer from MDD, which can lead to self-mutilation, suicidal tendencies, and harmful behaviors. It is estimated that by 2030, MDD will be the leading cause of disease burden worldwide [3, 4]. Sleep disturbance (SD) is a prominent symptom of MDD, affecting nearly two-thirds of patients with MDD during the course of illness [5]. Meta-analyses have shown that SD is positively correlated with the overall severity of MDD and its impact on quality of life [6, 7]. SD is also a risk factor for the onset and recurrence of MDD, increasing the risk of suicide [8]. However, the pathological mechanism of MDD with SD remains unclear.

Resting-state functional magnetic resonance imaging (rs-fMRI), an objective and noninvasive technology, has been widely used to explore the pathological mechanisms of mental disorders [9–11]. Regional homogeneity (ReHo) represents the temporal homogeneity of regional blood oxygen level-dependent signals and quantifies the temporal homogeneity of neural activities at rest. It has been utilized to investigate the pathological mechanisms underlying MDD and SD [12, 13]. Patients with MDD exhibit abnormal ReHo in the default-mode network (DMN) and cerebellum. Furthermore, abnormal ReHo in the left precuneus is positively correlated with SD scores in MDD patients [14, 15]. Patients with SD display abnormal ReHo in the frontal gyrus, cerebellum, occipital gyrus, and amygdala, and decreased ReHo in the occipital gyrus is negatively correlated with clinical symptoms of SD [16]. Resting-state functional connectivity between the bilateral amygdala and superior temporal gyrus is positively associated with SD scores in MDD patients [17]. Patients with MDD and SD exhibit abnormal temporal homogeneity of neural activities at rest. However, it is unclear whether MDD with SD has specific or distinctive alterations in ReHo and whether abnormal ReHo values can be used to distinguish MDD patients with SD.

Support vector machine (SVM) is a machine learning approach for multivariate pattern recognition that effectively defines a set of information and functions of different brain regions to find optimal separation hyperplanes in high-dimensional space for data classification [18, 19]. In SVM analysis, the optimal hyperplane is defined by finding the support vector [20]. Support vectors are data points closest to the hyperplane and play a crucial role in defining the position and orientation of the hyperplane [21]. SVM has great potential for predicting psychiatric disorders based on high-dimensional neuroimaging data [22–24]. Therefore, this study applied the SVM method to determine whether altered ReHo can identify sleep conditions in MDD patients.

In this study, we utilized ReHo and SVM methods to explore the pathological mechanisms underlying MDD patients with SD. We hypothesized that abnormal ReHo can be observed in certain brain regions in MDD patients with SD at rest and can be applied to identify sleep conditions in MDD patients.

## Materials and methods

### Participants

In the study, a total of sixty MDD patients and thirty-four age- and education-matched healthy controls (HCs) were included. All MDD patients were recruited from the psychiatric clinic of the Second Xiangya Hospital of Central South University. The diagnosis of MDD was performed by two trained senior psychiatrists following the criteria outlined in the fifth edition of Diagnostic and Statistical Manual of Mental Disorders (DSM-5). The severity of depression and anxiety was assessed using the 17-item Hamilton Rating Scale for Depression (HAMD-17) and Beck Anxiety Inventory (BAI). SD symptoms in each MDD patient were calculated based on the three items of the insomnia subscale (items four to six) of the HAMD-17 scale [25, 26]. Previous researches also defined SD based the items of the HAMD [27, 28]. Patients were stratified into MDD patients with SD (characterized by chief complaints of SD symptoms, and SD scores > 4) and MDD patients without SD (lacking chief complaints of SD symptoms, and SD scores ≤ 4) [29, 30]. All patients met the following criteria: ① aged between 18 and 55 years; ② right-handed; ③ first major depressive episode with HAMD-17 total scores > 20; ④ illness duration of at least 12 months; ⑤ no history of antipsychotics or electroconvulsive therapy; ⑥ no serious physical diseases, neurological disorder, or other psychiatric illness; ⑦ no drug or alcohol dependence; and ⑧ no contraindications for magnetic resonance imaging (MRI) scans. The HCs were enrolled from the community and screened using the SCID-I/NP (non-patient version).

The study was approved by the medical research ethics committee of the Second Xiangya Hospital of Central South University. All the procedures described herein comply with the Helsinki Declaration of 2013. Each participant provided informed consent before enrollment.

### Image acquisition and preprocessing

Resting-state functional images were acquired using a 3.0 T GE scanner (General Electric, Fairfield Connecticut, USA) at the Second Xiangya Hospital of Central South University. The echo planar imaging sequence was used to obtain images with the following parameters: TR, 2000 ms; TE, 30 ms; thickness, 4 mm; gap, 0.4 mm; FA, 90°; slices, 33; matrix, 64 × 64; and field of view (FOV), 220 mm × 220 mm. A total of 240 volumes were collected over a duration of 480 s.

Image preprocessing was performed using MATLAB toolboxes and Data Processing Assistant and Resting-State fMRI (DPARSF) [31, 32]. The preprocessing steps included discarding the first 10 functional volumes, slice timing correction, head motion correction, image normalization, spatial resampling to 3 × 3 × 3 mm^3^, bandpass filtering (0.01–0.08 Hz), and linear detrending.

### ReHo analysis

ReHo analyses were performed by calculating Kendall’s Coefficient of Concordance (KCC) values, which measure the synchronization of time series between a given voxel and its 26 adjacent voxels by using the REST software. The methodology for this approach has been described elsewhere [33]. To mitigate the effects of individual variations in KCC values, the ReHo map was standardized. Specifically, the KCC of each voxel was divided by the average KCC of the entire brain. Subsequently, the resulting ReHo maps underwent spatial smoothing with a Gaussian kernel of 4 mm FWHM. Finally, the smoothed ReHo maps were utilized for statistical analyses.

### Statistical analysis

Demographic and clinical data were analyzed using SPSS 22.0. Categorical variables, such as gender, were calculated by performing a chi-square test. Continuous variables, including illness duration, age, educational status, and scores of BAI, HAMD-17, and SD were analyzed using two-sample *t*-tests or one-way analysis of variance (ANOVA). The threshold for statistical significance was set at *p* < 0.05 (two tailed) for all tests.

The ReHo maps were compared using an analysis of covariance (ANCOVA) model. Post hoc *t*-tests were conducted to identify differences with age, gender, educational status, and mean framewise displacement (FD) values as covariates across the groups. The significance level was set at corrected *p* < 0.05 for multiple comparisons by using Gaussian Random Field theory (voxel significance: *p* < 0.001, cluster significance: *p* < 0.05). Since MDD patients with SD had higher total HAMD scores compared to MDD patients without SD, we reanalyzed the data with HAMD scores, age, gender, educational status, and mean FD values as covariates to minimize the confounding effects of depressive and SD symptoms.

Pearson/Spearman analysis was used to explore the correlations between abnormal ReHo values and scores of BAI, HAMD-17, and SD, with the Bonferroni correction for all patients, MDD patients with SD, MDD patients without SD, and HCs subjects, respectively.

### SVM analysis

SVM is widely employed in classification due to its proficiency in handling high-dimensional data and achieving high classification accuracy [34–36]. In this study, SVM analyses consisted of the following steps: 1) Obtained the dataset; 2) Data splitting: The entire dataset was divided into training and test datasets with a 0.5 ratio; 3) Feature normalization: Features were scaled to the range [0,1]; 4) Kernel selection: Gaussian radial basis function (RBF) kernels were chosen for classifier analysis. The RBF kernel features two parameters, ‘c’ and ‘g’; 5) Parameter optimization: A grid search method was employed for ‘c’ and ‘g’ via cross-validation to identify the optimal parameters; 6) Validation: To validate the SVM results, a 2-fold cross-validation method was applied. The dataset was divided into two equally sized subsets, and two classifier training sessions were conducted. In the first training session, one subset served as the training set, while the other acted as the test set. In the second training session, the training and test sets were swapped; 7) Performance metrics: Accuracy, sensitivity, and specificity were determined by summing the count of correct classifications in both the training and test sets.

**Fig. 1:**
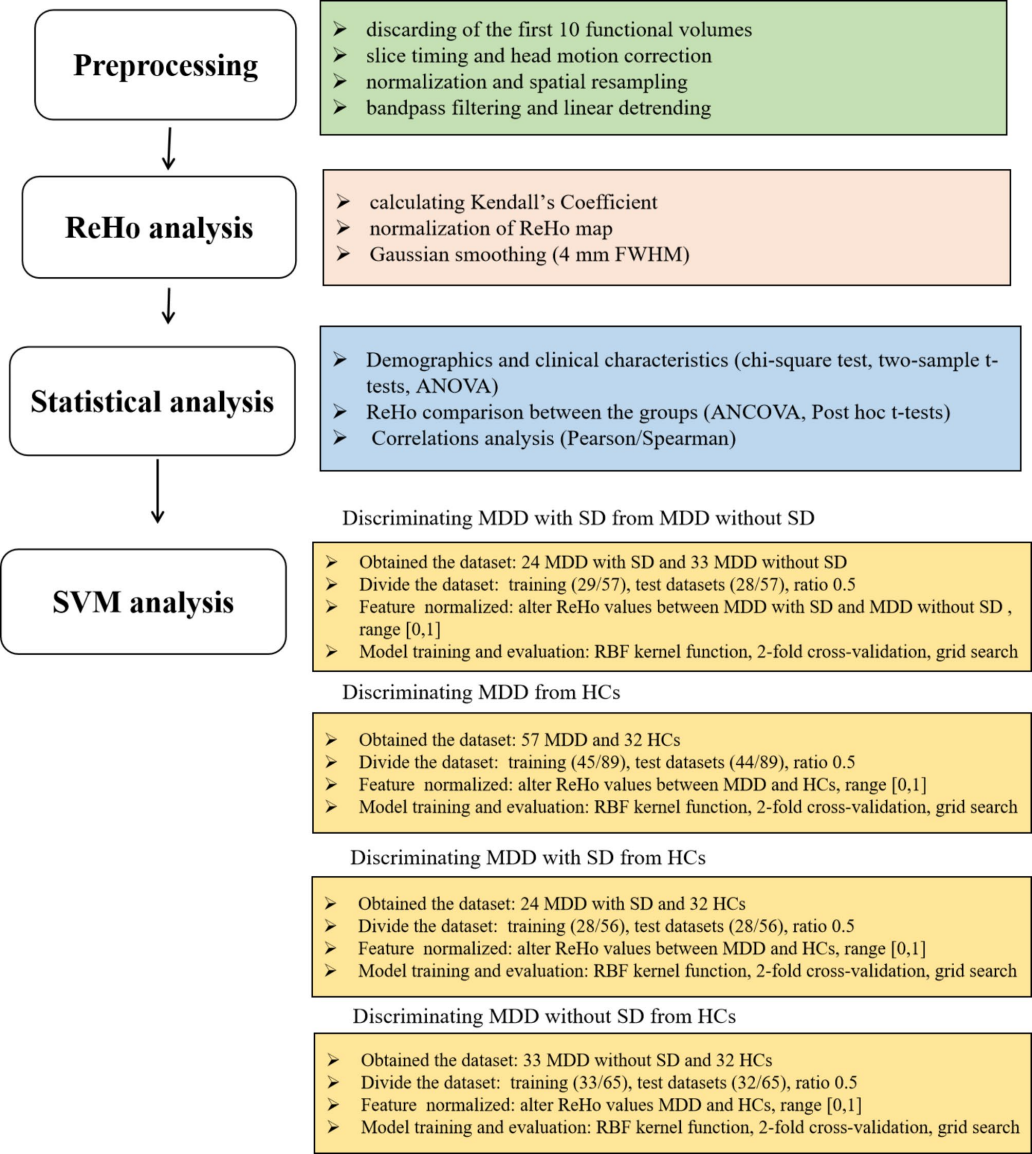
Flowchart of the analysis pipeline. Boxes on the left indicate general steps potentially applicable to a variety of data and analysis types; boxes on the right indicate particular choices made for the data and analysis presented here

Further details regarding the ReHo calculation process, statistical analysis, and SVM analysis were presented in Fig. 1.

## Results

### Demographics and clinical characteristics of participants

Five subjects were excluded due to excessive head movement (two MDD patients with SD, one MDD patient without SD, and two HCs). Eventually, a total of 24 MDD patients with SD, 33 MDD patients without SD, and 32 HCs were included in our study. There were no differences in age and education status among the three groups except for gender, and no difference in illness duration between the two MDD groups. The MDD with SD group showed higher BAI scores, HAMD-17 total scores, and SD scores than the MDD without SD group. Both MDD groups showed higher scores in anxiety/somatization, retardation symptoms, weight loss, and cognitive disturbance than HCs. However, no significant differences were found in these aforementioned features between the MDD with SD group and MDD without SD group (Table 1).

**Table 1:** Demographic and clinical characteristics of participants

Variables	*Pa_s* group (n = 24)	*Pa_ns* group (n = 33)	HCs (n = 32)	*F*/χ^2^/*t*	*Post hoc t-tests or p/t values*
Age (years)	31.375 ± 6.78	29.48 ± 7.13	29.59 ± 5.00	1.07^a^	0.35
Gender (male/female)	12/12	6/27	15/17	8.09^b^	0.02
Education (years)	13.63 ± 3.73	13.91 ± 3.06	14.59 ± 2.82	0.72^a^	0.49
Illness duration (months)	5.83 ± 4.12	6.77 ± 4.65		0.78^c^	0.43
BAI -	47.39 ± 13.11	37.97 ± 7.58	22.63 ± 2.28	63.75^a^	*Pa_s* > *Pa_ns* > HCs
HAMD − 17 scores	23.38 ± 3.70	20.18 ± 2.64	0.94 ± 0.95	670.29^a^	*Pa_s* > *Pa_ns* > HCs
Sleep disturbances^*^	5.54 ± 0.51	3.15 ± 0.94	0.34 ± 0.60	357.41^a^	*Pa_s* > *Pa_ns* > HCs
Anxiety/Somatization	7.38 ± 1.91	6.76 ± 1.82	0.44 ± 0.62	190.43^a^	*Pa_s*, *Pa_ns* > HCs
Retardation symptoms	6.25 ± 1.51	6.64 ± 1.32	0.16 ± 0.37	313.83^a^	*Pa_s, Pa_ns* > HCs
Weight loss	0.71 ± 0.81	0.39 ± 0.70	0	9.83^a^	*Pa_s, Pa_ns* > HCs
Cognitive disturbances	3.50 ± 2.04	3.24 ± 1.70	0	52.83^a^	*Pa_s, Pa_ns* > HCs

Data was displayed with mean ± standard deviation. HAMD-17, the 17-item Hamilton Rating Scale for Depression; BAI, Beck anxiety inventory; *Pa_s*, major depressive disorder with sleep disturbances; *Pa_ns*, major depressive disorder without sleep disturbances; HCs, healthy controls

^a^ ANOVA

^b^ Chi-square test

^c^ Two sample *t*-test

^*^Sleep disturbance scores were computed by the fourth to sixth items of the HAMD-17 scale

### Differences in ReHo between groups

**Fig. 2:**
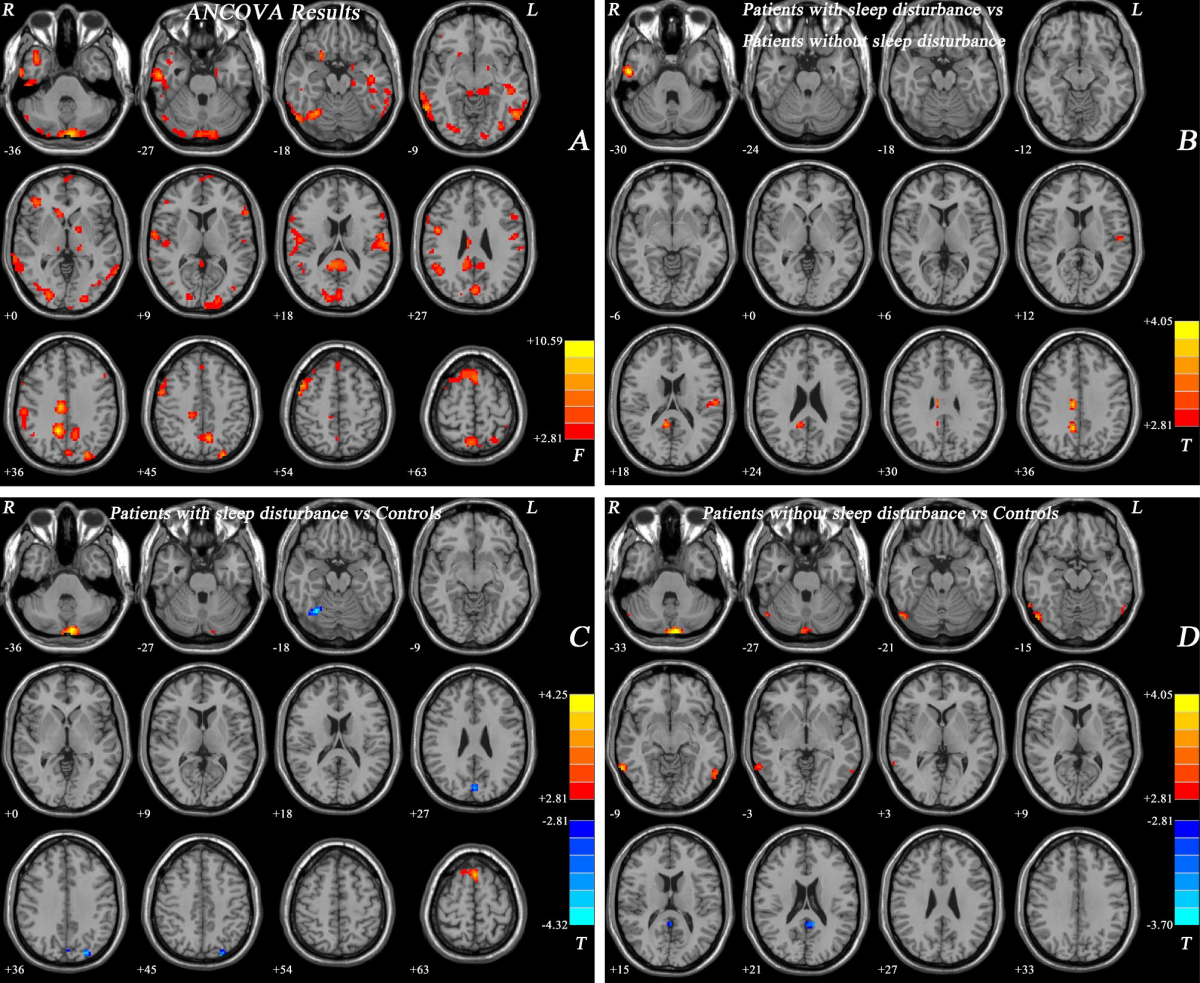
(**A**) Brain regions with abnormal ReHo in the three groups based on ANCOVA with the covariates of age, gender, educational status, and mean FD values. Red colors denote significant difference among groups. The color bar indicates the *F* value from ANCOVA. (**B**) Brain regions with abnormal ReHo between MDD with SD and MDD without SD based on post-hoc *t*-tests. Red colors denote high ReHo in MDD with SD. The color bar indicates the T value. (**C**) Brain regions with abnormal ReHo between MDD with SD and HCs based on post hoc *t*-tests. Red colors denote high ReHo, and blue colors denote low ReHo in MDD with SD. The color bars indicate the T value. (**D**) Brain regions with abnormal ReHo between MDD without SD and HCs based on post hoc *t*-tests. Red colors denote high ReHo, and blue colors denote low ReHo in MDD without SD. The color bars indicate the T value. ReHo, regional homogeneity; SD, sleep disturbance; ANCOVA, analysis of covariance; FD, framewise displacement; MDD, major depressive disorder; HCs, healthy controls

According to ANCOVA analysis, significant changes in ReHo values were observed in the temporal, occipital, frontal, cerebellar, and limbic regions for the three groups (Fig. 2A).

In comparison to MDD patients without SD, MDD patients with SD exhibited increased ReHo values in the right posterior cingulate cortex (PCC)/precuneus, right median cingulate cortex (MCC), right inferior temporal gyrus (ITG) and left postcentral gyrus (postCG) (Fig. 2B; Table 2).

**Table 2:** Significant ReHo differences across three groups

Cluster location	Peak (MNI)			Number of voxels	*T* value
	x	y	z		
***Pa_s vs. Pa_ns***					
Right PCC/precuneus	9	-51	36	72	3.8734
Right MCC	9	-21	36	39	3.8839
Right ITG	60	-12	-30	47	4.1982
Left postCG	-57	-18	15	35	3.5905
***Pa_s vs. HCs***					
Bilateral Cerebellum Crus2	-9	-90	-33	94	4.2532
Bilateral MFG	0	21	63	75	3.8023
Right Fusiform Gyrus/Cerebellum 6	30	-63	-15	38	-4.3164
Left Cuneus	0	-81	27	37	-3.7039
Left SOG	-24	-84	39	36	-4.3143
***Pa_ns vs. HCs***					
Bilateral Cerebellum Crus2	-6	-93	-33	75	4.0532
Right MOG	54	-72	-18	49	3.6440
Left ITG	-63	-60	-12	35	3.3091
Right MTG	66	-57	-9	45	3.7689
Bilateral PCC/precuneus	-9	-45	21	32	-3.3397

MNI, Montreal Neurological Institute; ReHo, regional homogeneity. *Pa_s*, major depressive disorder with sleep disturbances; *Pa_ns*, major depressive disorder without sleep disturbances; HCs, healthy controls; PCC, posterior cingulate cortex; MCC, median cingulate cortex; ITG, inferior temporal gyrus; postCG, postcentral gyrus; MFG, medial frontal gyrus; SOG, superior occipital gyrus; MOG, middle occipital gyrus; MTG, middle temporal gyrus

In comparison to HCs, MDD patients with SD exhibited increased ReHo values in the bilateral cerebellum crus 2 and bilateral medial frontal gyrus (MFG), and decreased ReHo in the right fusiform gyrus/cerebellum crus 6, left cuneus, and left superior occipital gyrus (SOG) (Fig. 2C; Table 2).

In comparison to HCs, MDD patients without SD displayed increased ReHo in the bilateral cerebellum crus 2, right middle occipital gyrus (MOG), left ITG, and right middle temporal gyrus (MTG) and decreased ReHo in the bilateral PCC/precuneus (Fig. 2D; Table 2).

In comparison to HCs, all MDD patients showed increased ReHo values in the bilateral cerebellum crus 2, right MFG and right ITG, and decreased ReHo in the right MOG (Table S1).

These results remained consistent when considering HAMD scores, age, gender, educational status, and mean FD values as covariates (Table S2).

### Correlations between ReHo and clinical characteristics

**Fig. 3:**
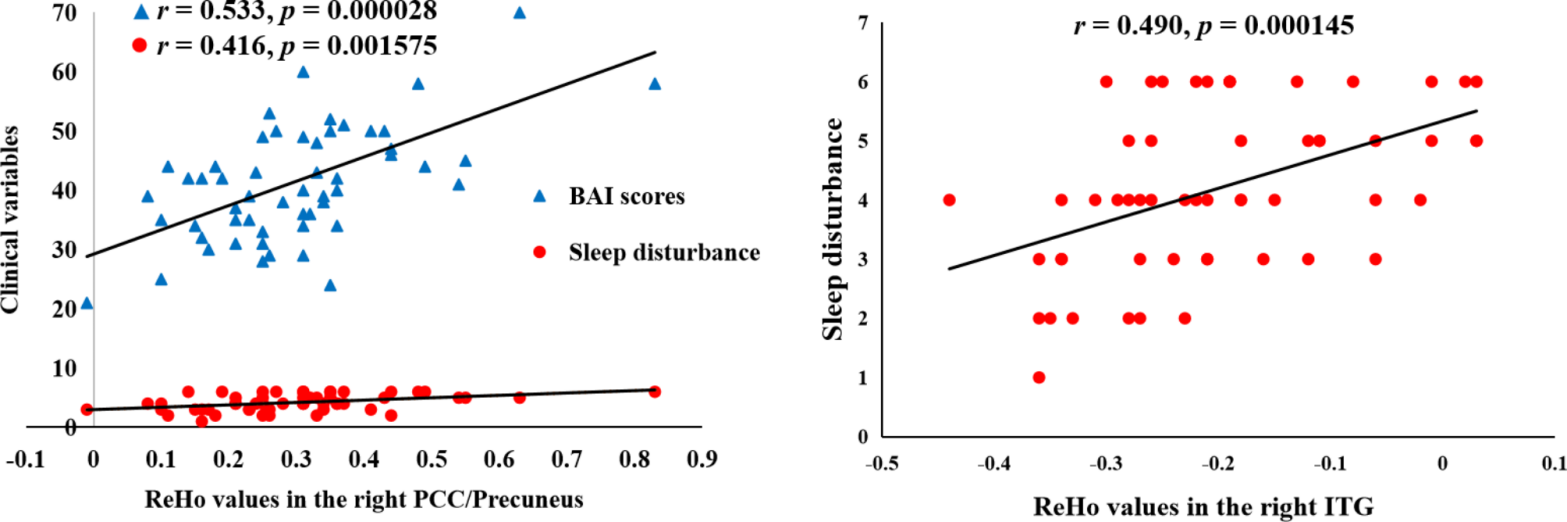
Correlations between ReHo values and clinical variables. For all patients with MDD, increased ReHo values of the right PCC/precuneus were positively correlated with the scores of BAI and SD (Left). For all patients with MDD, increased ReHo of the right ITG was positively correlated with the SD scores (Right). ReHo, regional homogeneity; BAI, Beck anxiety inventory; PCC, posterior cingulate cortex; ITG, inferior temporal gyrus

For all patients, increased ReHo values in the right PCC/precuneus were positively correlated with the total scores of BAI (*r* = 0.533, *p* = 0.000028) and SD (*r* = 0.416, *p* = 0.001575), and increased ReHo in the right ITG was positively correlated with the SD scores (*r* = 0.490, *p* = 0.000145) (Fig. 3). Abnormal ReHo values were not correlated with BAI, HAMD-17, and SD scores in HC subjects or in MDD patients with or without SD.

### SVM results

#### Discriminating MDD patients with SD from MDD patients without SD

**Fig. 4:**
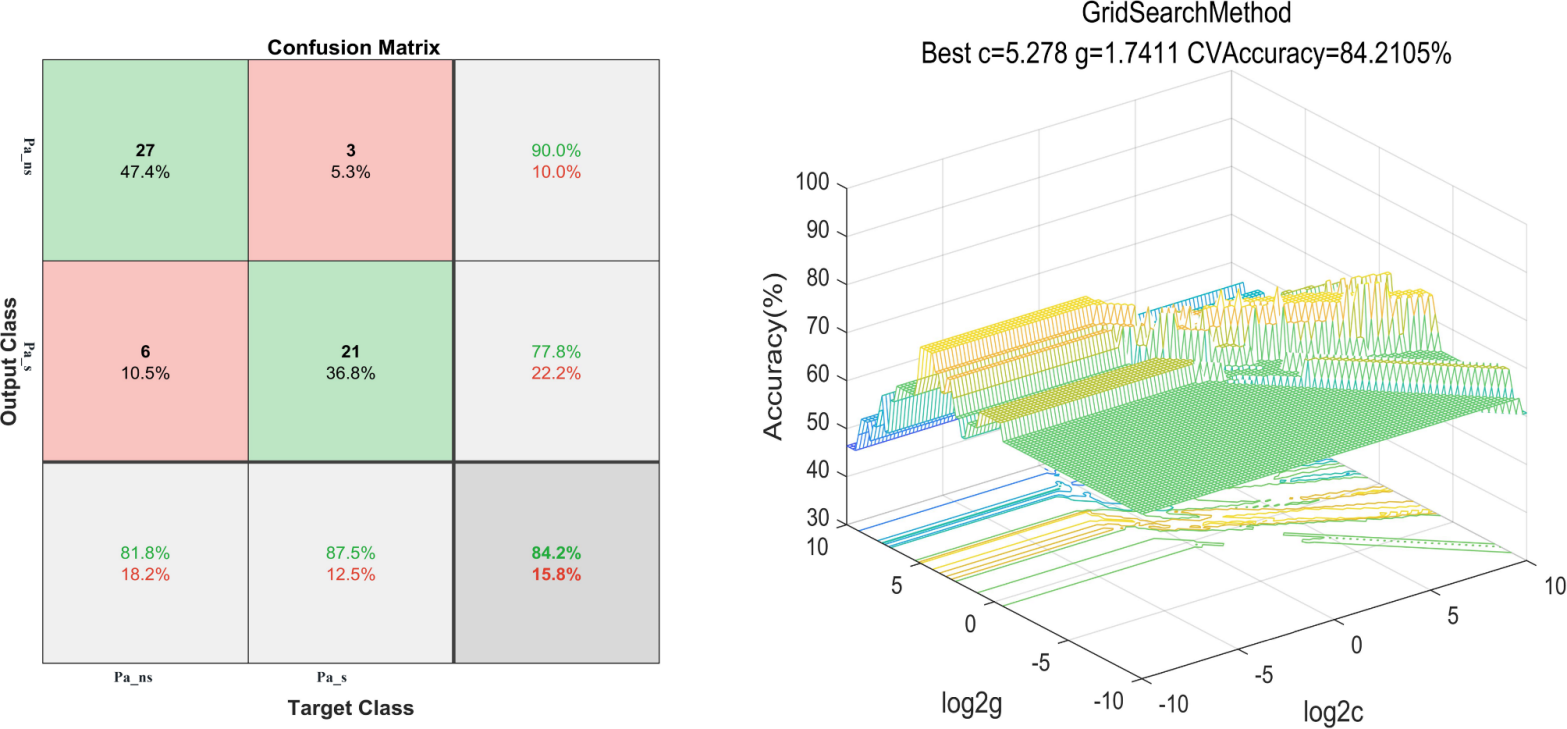
Visualization of classifications through SVM using the combination of ReHo values in the right ITG and left postCG (features 3 and 4) to discriminate MDD with SD and MDD without SD. Left: Confusion matrix map of the combination of ReHo values in the right ITG and left postCG. The target class conveys the correct classification of each subject. The output class conveys the predicted classification of each subject. Red boxes represent incorrect predictions, and green boxes represent correct predictions. Right: 3D visualization of SVM with the best parameters. Log 2c and log 2 g mean the range and step size of the given parameters c and g (c and g are the parameters of the kernel functions in SVM training). ReHo, regional homogeneity. Pa_s, major depressive disorder with sleep disturbances; Pa_ns, major depressive disorder without sleep disturbances; SVM, support vector machine; postCG, postcentral gyrus; ITG, inferior temporal gyrus

Abnormal ReHo between MDD patients with SD and MDD patients without SD represented as feature variables (1 = right PCC/precuneus, 2 = right MCC, 3 = right ITG, 4 = left postCG), which were entered into the classification models. The combination of the ReHo values of 3 and 4 (Table 3; Fig. 4) could optimally discriminate MDD patients with SD from those without SD with accuracy, sensitivity, and specificity rates of 84.21% (48/57), 87.50% (21/24), and 81.82% (27/33), respectively.

**Table 3:** The results of SVM analysis based on the selected optimal features

Features	Accuracy (%)	Sensitivity (%)	Specificity (%)
***Pa_s vs. Pa_ns***			
Combine 3 and 4	84.21 (48/57)	87.50 (21/24)	81.82 (27/33)
3 = right ITG			
4 = left postCG			
***All MDD Patients vs. HCs***			
Combine 1, 2 and 4	80.90 (72/89)	94.74 (54/57)	56.25 (18/32)
1 = bilateral cerebellum crus2			
2 = right MFG			
4 = right ITG			

*Pa_s*, major depressive disorder with sleep disturbances; *Pa_ns*, major depressive disorder without sleep disturbances; MDD, major depressive disorder; HCs, healthy controls; ITG, inferior temporal gyrus; postCG, postcentral gyrus; MFG, medial frontal gyrus

#### Discriminating MDD patients from HCs

**Fig. 5:**
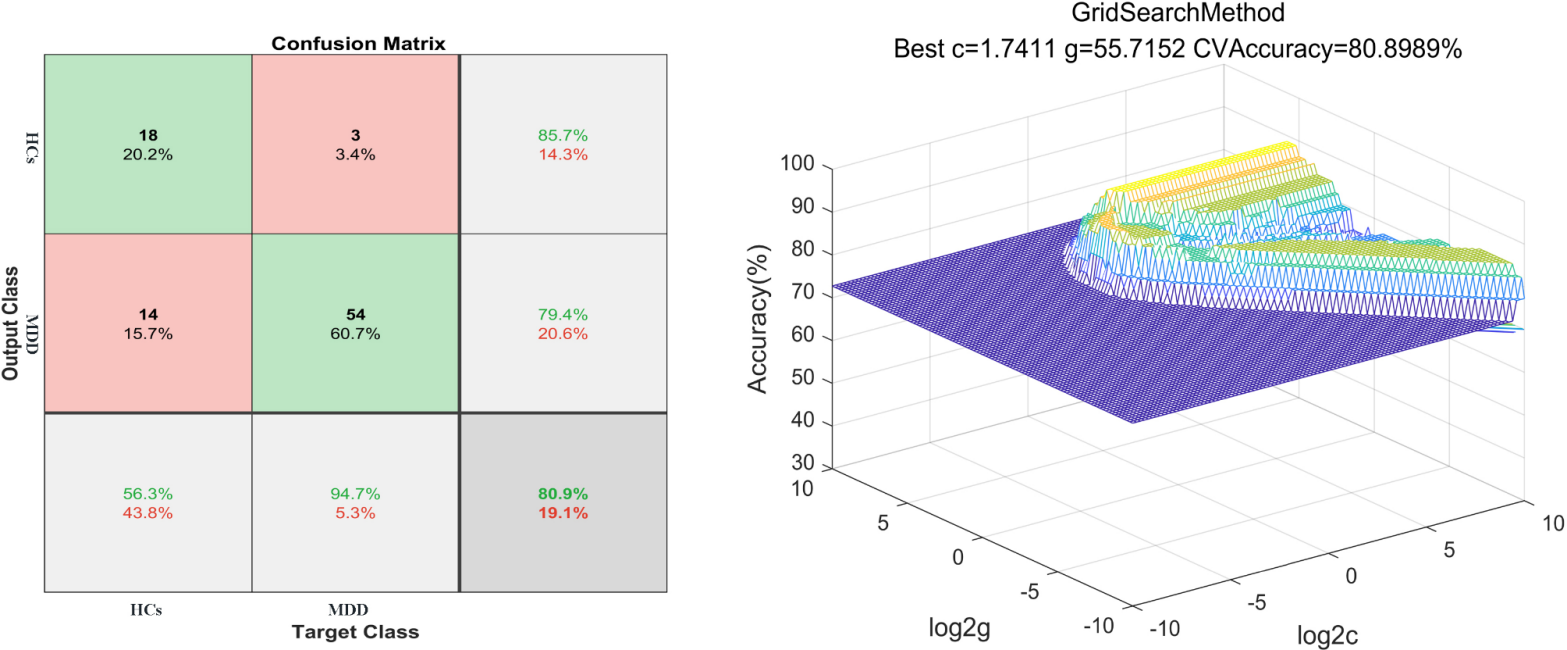
Visualization of classifications through SVM using the combination of ReHo values in the bilateral cerebellum crus 2, right MFG and right ITG (features 1, 2 and 4) to discriminate MDD patients and HCs. Left: Confusion matrix map of the combination of ReHo values in the bilateral cerebellum crus 2, right MFG and right ITG. The target class conveys the correct classification of each subject. The output class conveys the predicted classification of each subject. Red boxes represent incorrect predictions, and green boxes represent correct predictions. Right: 3D visualization of SVM with the best parameters. Log 2c and log 2 g mean the range and step size of the given parameters c and g (c and g are the parameters of the kernel functions in SVM training). MDD, major depressive disorder; SD, sleep disturbance; HCs, healthy controls; ReHo, regional homogeneity; SVM, support vector machine; MFG, medial frontal gyrus; ITG, inferior temporal gyrus

Abnormal ReHo between MDD patients and HCs (Table S1) represented as feature variables (1 = bilateral cerebellum crus2, 2 = right MFG, 3 = right MOG, 4 = right ITG), which were entered into the classification models. The combination of the ReHo values of 1, 2 and 4 exhibited a high sensitivity (94.74%) and a low specificity (56.25%) in discriminating MDD patients from HCs (Table 3; Fig. 5). To provide a clearer understanding of the high sensitivity and low specificity in distinguishing MDD patients from HCs, we conducted SVM analyses using altered ReHo values between MDD and HCs to differentiate MDD patients with SD from HCs, and MDD patients without SD from HCs. The SVM results showed that the combination of ReHo values of 1, 2 and 4 achieved a good sensitivity of 75.00% and a specificity of 78.79% in differentiating MDD patients with SD from HCs. Similarly, these same regions in the brain could distinguish MDD patients without SD from HCs with a good sensitivity of 81.25% and a specificity of 75.00% (Table S3, Fig. S1 and Fig. S2).

## Discussion

The results revealed that MDD patients with SD exhibited increased ReHo in the right PCC/precuneus, right MCC, right ITG, and left postCG compared with MDD patients without SD. In addition, increased ReHo values of the right PCC/precuneus and ITG were positively correlated with the SD scores. A combination of ReHo in the left postCG and right ITG can be utilized in distinguishing MDD patients with SD from those without SD, showing optimal specificity and sensitivity. These findings provide insight for further clinical diagnosis and syndrome sub-classification.

MDD patients with SD exhibited increased ReHo in the right PCC/precuneus and right MCC compared with MDD patients without SD, and increased ReHo in the right PCC/precuneus showed a positive relationship with the SD scores. PCC/precuneus and MCC are important brain regions of the DMN and are generally related to negative self-focus and disturbed emotional regulation in patients with MDD [37, 38]. The rates of volume loss in the right PCC were negatively associated with sleep quality, suggesting that poor sleep quality significantly accelerated volume loss in the right PCC [39]. Impaired sleep in patients with MDD was associated with increased connectivity in the function of the DMN, which includes regions responsible for self-reflection and emotional processing [40, 41]. Consistent with previous findings, increased ReHo values in PCC/precuneus and MCC within the DMN were involved in the compensatory response to emotional regulation and self-perceptions in MDD patients with SD, which might lead to difficulty in falling asleep and poor sleep quality [42].

As a component of the auditory cortex, ITG exhibits decreased functional connectivity in responses to external stimuli during sleep [17]. Stimulation related to external auditory stimuli leads to increased responsiveness to insomnia [43]. The present research showed increased ReHo in the ITG and a positive correlation with SD scores in MDD patients with SD, suggesting that ITG is associated with high arousal status in these patients, who were sensitive to external auditory stimuli during sleep. Patients with primary insomnia showed progressively increased gray matter volume in the right ITG [44]. Furthermore, the correlation between psychological stress and sleep quality may be mediated by the bilateral ITG [45]. These findings highlight that the increased regional neural activity of the ITG may be involved in the pathophysiological mechanism underlying SD in patients with MDD [28].

As a key brain area of the somatosensory network, the postCG plays an important role in sensory–motor integration and transmission [46, 47]. Compared with MDD patients without SD, MDD patients with SD showed increased ReHo value in the left postCG in the current study. A previous research found that reduction in the gray matter volume of the left postCG is related to the severity of SD and depressive symptom in shift-working nurses [48]. Congruent with previous findings, we suggest that increased ReHo in the left postCG leads to excessive sensory–motor information integration through the activation of the somatosensory network, thereby affecting sleep sensitivity in patients with MDD [49]. SVM analysis results showed a combination of increased ReHo values in the left postCG and right ITG exhibits the highest accuracy (84.21%) in discriminating MDD patients with SD from those without SD. Thus, we suggest that increased ReHo values in the left postCG and right ITG can be used as a potential neurobiological marker for MDD patients with SD. Furthermore, SVM results showed that the combination of ReHo values in the bilateral cerebellum crus 2, right MFG and right ITG exhibited a high sensitivity (94.74%) and low specificity (56.25%) in discriminating MDD patients from HCs. However, the combination of ReHo values of these same regions achieved a good sensitivity of 75.00% and specificity of 78.79% in differentiating MDD patients with SD from HCs. Similarly, these same regions in the brain could distinguish MDD patients without SD from HCs with a good sensitivity of 81.25% and specificity of 75.00%. These findings indicate that increased ReHo values in the bilateral cerebellum crus 2, right MFG and right ITG could be utilized for future MDD classification. The initial observation of high sensitivity and low specificity in distinguishing MDD patients from HC may be attributed to class size imbalance (MDD: 57 vs. HCs: 32).

Additionally, compared with HCs, increased ReHo values in the bilateral cerebellum crus 2 were found in MDD patients with and without SD. The cerebellum crus 2 was the intersection of the two subtypes of MDD, indicating that cerebellum crus 2 is involved in the pathological mechanism of MDD patients with and without SD. Compared with HCs, MDD patients with SD showed increased ReHo values in the bilateral MFG and decreased ReHo in the right fusiform gyrus/cerebellum crus 6, left cuneus, and left SOG. However, the MDD patients without SD displayed increased ReHo in the right MOG, left ITG, and right MTG, and decreased ReHo in the bilateral PCC/precuneus. These findings suggest that different subtypes of MDD have diverse neuropathological mechanisms.

Several limitations deserve to be mentioned. First, the sample size may limit the statistical power in detecting subtle brain alterations and uncovering potential depression–brain–sleep relationship. Second, refined classification of SD was not included in our current study, such as difficulties and quality of sleep duration and sleep fragmentation. Third, most patients with MDD were adults, and the current findings may not generalize to adolescent patients with MDD. Finally, the SD symptoms of each patient with MDD were calculated according to the three items insomnia subscale of the HAMD-17 scale. However, this is not robust, and future studies should utilized valid sleep questionaries such as the Pittsburgh Sleep Quality Index (PSQI) for the assessment of SD.

## Conclusions

Our present study addressed the specific or distinctive ReHo patterns in MDD patients with SD. Increased ReHo in the right ITG and PCC/precuneus might represent stable and unique neurobiological features of MDD patients with specific sleep conditions. In addition, a combination of abnormal ReHo in the postCG and right ITG may be applied as a potential neurobiological marker for discriminate MDD patients with SD from those without SD.

### Electronic supplementary material

Below is the link to the electronic supplementary material.

Supplementary Material 1

## Data Availability

The data may be available from the corresponding author upon reasonable request.

## Abbreviations

MDD: Major Depressive Disorder
HCs: Healthy Controls
SD: Sleep Disturbance
rs-fMRI: Resting state functional magnetic resonance imaging
ReHo: Regional homogeneity
DMN: Default-mode network
SVM: Support vector machine
HAMD-17: 17-item Hamilton Rating Scale for Depression
BAI: Beck Anxiety Inventory
MRI: Magnetic resonance imaging
DPARSF: Data Processing Assistant and Resting-State fMRI
KCC: Kendall’s Coefficient of Concordance
ANOVA: Analysis of variance
GRF: Gaussian Random Field
FD: Framewise displacement
PCC: Posterior cingulate cortex
MCC: Median cingulate cortex
postCG: Postcentral gyrus
ITG: Inferior temporal gyrus
MFG: Medial frontal gyrus
SOG: Superior occipital gyrus
MOG: Middle occipital gyrus
MTG: Middle temporal gyrus
