# Supplementary material for: Identifying major depressive disorder with associated sleep disturbances through fMRI regional homogeneity at rest
Source: BMC Psychiatry. 2023 Nov 7;23:809. doi: 10.1186/s12888-023-05305-7 (PMC10631123; doi:10.1186/s12888-023-05305-7)
Supplement: Supplementary file 1 — Supplementary Material 1 [file 12888_2023_5305_MOESM1_ESM.docx]

**Supplementary materials**

**Materials and methods**

**Statistical analysis**

The ReHo values were compared between two groups (All MDD Patients vs HCs) through voxel-wise two-sample *t*-test by taking age, gender, educational status, and mean FD values as covariates. The significant threshold was *P*<0.05 (Gaussian random field corrected, voxel *p*<0.001, cluster *P*<0.05).

**Results**

**Differences in ReHo between MDD Patients and HCs**

Compared with HCs, All MDD patients showed increased ReHo values in the bilateral cerebellum crus 2, right MFG and right ITG, and decreased ReHo in the right MOG (Table S1).

**Table S1** Significant ReHo differences between all MDD patients and HCs.

| Cluster location | Peak (MNI) | | | Number of voxels | *T* value |
| --- | --- | --- | --- | --- | --- |
|  | x | y | z |  |  |
| *All MDD Patients vs HCs* |  |  |  |  |  |
| Bilateral Cerebellum Crus2 | -9 | -90 | -33 | 112 | 4.3819 |
| Right MFG | 51 | 9 | 54 | 31 | 3.5215 |
| Right MOG | 24 | -87 | -3 | 32 | -3.4483 |
| Right ITG | 63 | -54 | -9 | 44 | 3.6760 |

MNI, Montreal Neurological Institute; ReHo, regional homogeneity; MDD, major depressive disorder; HCs, healthy controls; MFG, medial frontal gyrus; MOG, middle occipital gyrus; ITG, inferior temporal gyrus.

**Tables S2** Significant ReHo differences between *Pa_s* and *Pa_ns* with the HAMD scores, age, gender, educational status, and mean FD values as covariates

| Cluster location | Peak (MNI) | | | Number of voxels | *T* value |
| --- | --- | --- | --- | --- | --- |
|  | x | y | z |  |  |
| *Pa_s vs* *Pa_ns* |  |  |  |  |  |
| Right PCC/precuneus | 9 | -54 | 36 | 82 | 3.8642 |
| Right MCC | 9 | -21 | 36 | 49 | 4.0420 |
| Right ITG | 60 | -9 | -30 | 33 | 3.9555 |
| Left postCG | -57 | -18 | 15 | 22 | 3.4727 |

MNI, Montreal Neurological Institute; ReHo, regional homogeneity. *Pa_s*, major depressive disorder with sleep disturbances; *Pa_ns*, major depressive disorder without sleep disturbances; PCC, posterior cingulate cortex; MCC, median cingulate cortex; ITG, inferior temporal gyrus; postCG, postcentral gyrus.

**Table** **S3** The results of SVM analysis based on the selected optimal features.

| Features | Accuracy (%) | Sensitivity (%) | Specificity (%) |
| --- | --- | --- | --- |
| ***Pa_s vs HCs*** |  |  |  |
| Combine 1, 2 and 4 | 78.57 (44/56) | 75.00 (18/24) | 81.25 (26/32) |
| 1 = bilateral cerebellum crus2 |  |  |  |
| 2 = right MFG |  |  |  |
| 4 = right ITG |  |  |  |
|  |  |  |  |
| ***Pa_ns vs HCs*** |  |  |  |
| Combine 1, 2 and 4 | 76.92 (50/65) | 78.79 (26/33) | 75.00 (24/32) |
| 1 = bilateral cerebellum crus2 |  |  |  |
| 2 = right MFG |  |  |  |
| 4 = right ITG |  |  |  |

*Pa_s*, major depressive disorder with sleep disturbances; *Pa_ns*, major depressive disorder without sleep disturbances; HCs, healthy controls; ITG, inferior temporal gyrus; MFG, medial frontal gyrus.


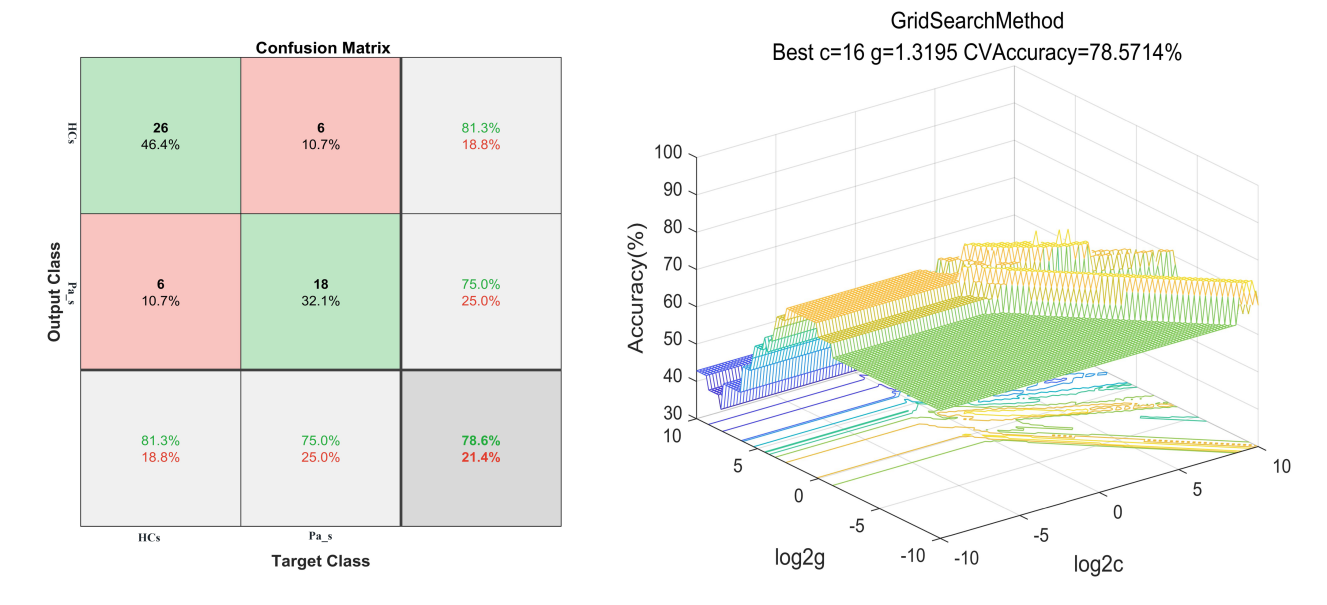


**Fig. S1** Visualization of classifications through SVM using the combination of ReHo values in the bilateral cerebellum crus 2, right MFG and right ITG (features 1, 2 and 4) to discriminate MDD patients with SD and HCs. Left: Confusion matrix map of the combination of ReHo values in the bilateral cerebellum crus 2, right MFG and right ITG. The target class conveys the correct classification of each subject. The output class conveys the predicted classification of each subject. Red boxes represent incorrect predictions, and green boxes represent correct predictions. Right: 3D visualization of SVM with the best parameters. Log 2c and log 2g mean the range and step size of the given parameters c and g (c and g are the parameters of the kernel functions in SVM training). Pa_s, major depressive disorder with sleep disturbances; MDD, major depressive disorder; SD, sleep disturbance; HCs, healthy controls; ReHo, regional homogeneity; SVM, support vector machine; MFG, medial frontal gyrus; ITG, inferior temporal gyrus.


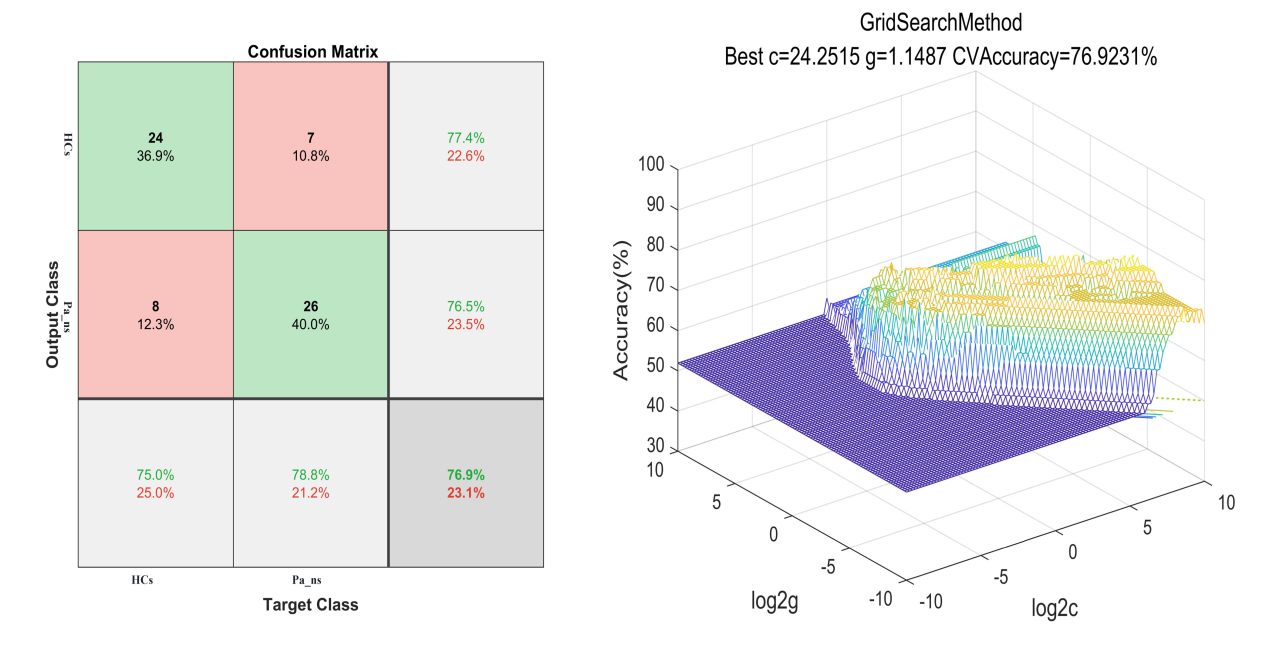


**Fig. S2** Visualization of classifications through SVM using the combination of ReHo values in the bilateral cerebellum crus 2, right MFG and right ITG (features 1, 2 and 4) to discriminate MDD patients without SD and HCs. Left: Confusion matrix map of the combination of ReHo values in the bilateral cerebellum crus 2, right MFG and right ITG. The target class conveys the correct classification of each subject. The output class conveys the predicted classification of each subject. Red boxes represent incorrect predictions, and green boxes represent correct predictions. Right: 3D visualization of SVM with the best parameters. Log 2c and log 2g mean the range and step size of the given parameters c and g (c and g are the parameters of the kernel functions in SVM training). Pa_ns, major depressive disorder without sleep disturbances; MDD, major depressive disorder; SD, sleep disturbance; HCs, healthy controls; ReHo, regional homogeneity; SVM, support vector machine; MFG, medial frontal gyrus; ITG, inferior temporal gyrus.
